# Decision-making for termination of pregnancy following non-invasive prenatal testing: a qualitative exploration of french, english and German healthcare professionals’ perceptions and concerns

**DOI:** 10.1186/s12978-025-02120-z

**Published:** 2025-11-03

**Authors:** Adeline Perrot, Hilary Bowman-Smart, Tamar Nov-Klaiman, Ruth Horn

**Affiliations:** 1 Nuffield Department of Population Health, Ethox Centre, University of Oxford, Oxford, UK; 2 Australian Centre for Precision Health, University of South Australia, Adelaide, Australia; 3 Biomedical Ethics Research Group, Murdoch Children’s Research Institute, Melbourne, Australia; 4 Institute of Ethics and History of Health in Society, University of Augsburg, Augsburg, Germany

**Keywords:** Prenatal screening, Termination of pregnancy, Informed choice, Down’s syndrome, Decision-making, Reproductive autonomy, France, England and Germany

## Abstract

Non-invasive prenatal testing (NIPT) is offered in the French, English and German public healthcare systems for fetal aneuploidy screening from a relatively early stage of pregnancy (around 10–12 weeks of gestation). Results from NIPT can be used to inform decisions about subsequent diagnostic procedures, and pregnancy management, which may include options for termination of pregnancy (TOP). Since NIPT is a screening test and not diagnostic, clinical guidelines recommend confirmation through an invasive procedure. Across the three countries, healthcare professionals (HCPs) expressed concerns that women may make uninformed decisions around TOP, in the sense of not being fully informed either regarding the test performance or regarding the fetal condition. This paper draws on a comprehensive literature review, and data from a comparative study including 58 semi-structured interviews with French, English and German healthcare professionals (HCPs) on ethical issues, perceptions and regulations regarding NIPT and TOP. HCPs in our study expressed a number of fears and concerns about how and why women or prospective parents might make uninformed decisions about TOP. Their key concerns include decisions being made based on a NIPT result without diagnostic confirmation, with references to anecdotal accounts, and biased or uninformed perceptions of genetic conditions such as Down’s Syndrome. Our findings highlight how the desire for autonomy in pregnancy decisions may in some ways conflict with HCPs’ duty to ensure that counselling takes place, and that women or prospective parents have an adequate level of understanding of the implications of NIPT.

## Introduction

Non-invasive prenatal testing (NIPT) is offered in the French, English and German public healthcare systems for fetal aneuploidy screening from a relatively early stage of pregnancy (10–12 weeks of gestation). NIPT panels generally include trisomies 21, 13 and 18 (Down’s syndrome, Patau’s syndrome and Edward’s syndrome), but some regions or providers may also include an expanded offer covering other aneuploidies or genetic conditions [1]. Results from NIPT can be used to inform decisions about subsequent diagnostic procedures, and pregnancy management, which may include options for termination of pregnancy (TOP). Informed choice and reproductive autonomy are frequently positioned as central in policies and clinical practices relating to prenatal testing and selective TOP [2, 3]. This sits alongside ‘non-directiveness’ as a key value in genetic counselling more broadly, which may be offered for pregnancies where a fetal diagnosis has been made [4]. Significant attention is thus paid in the bioethics literature to both the ethics of selective TOP [5, 6], as well as how to best support and understand reproductive autonomy in the prenatal setting [7].

However, this ‘rhetoric of choice’ has also been critiqued for failing to recognise how the social context and institutional aspects of service delivery of prenatal screening can affect choice at the level of the individual [6, 8]. The non-invasive nature of NIPT compared to invasive diagnostic tests like amniocentesis may shape moral reasoning and attitudes [9, 10] during pregnancy. For example, where women or prospective parents decide not to use prenatal screening technologies, a decreased risk of harm to the fetus can make an explanation or rationale more difficult to articulate. While those who undergo NIPT may generally have a good understanding of the test characteristics, it is also often described as ‘easy’ or ‘just a blood test’ [11]. Decision-making processes draw on a wide range of information beyond technical information about the test, and include individual, contextual and relational factors [12]. However, studies from the UK, Canada, China, Denmark, and the Netherlands suggest that decisional conflict and decision regret regarding NIPT is generally low, though this may be higher in certain groups – e.g. those who have received a false-positive result [13].

After the receipt of a NIPT test result or prenatal diagnosis, prospective parents may then be faced with a further set of choices for pregnancy management (prenatal intervention, planning the birth procedure and care of the future child), which includes options relating to TOP. As NIPT is a screening test and not diagnostic, clinical guidelines across the world recommend confirmation through an invasive procedure such as CVS or amniocentesis [14]. However, concerns have been raised in the literature, and anecdotal cases reported in the media, of women or prospective parents choosing TOP based on a NIPT result without diagnostic confirmation [15, 16]. While the evidence for and extent of this phenomenon is unclear, restrictive or changing TOP legislation may impact this. In the United States, after the overturning of Roe v Wade, many states have introduced strict gestational limits for TOP access; this may result in a reliance on screening results for decision-making because it may not be possible to get diagnostic confirmation within these gestational limits [17].

In this paper, we draw on a cross-cultural comparative qualitative interview study between France, England and Germany to explore healthcare professionals’ (HCPs’) perspectives and concerns about the information and factors that women use to make decisions about TOP following a NIPT result. There is a range of empirical research in the literature on NIPT exploring perspectives on informed choice, but these studies are primarily conducted with women or prospective parents rather than HCPs [18]. This paper draws on a previous comprehensive literature review, and data from a comparative study including 58 semi-structured interviews with French, English and German HCPs on ethical issues, perceptions and regulations regarding NIPT and TOP. Furthermore, this work builds on a previous manuscript which explored professional values in the counselling process, and physician-patient dynamic [3].

HCPs in our study expressed a number of fears and concerns about how and why women or prospective parents might make uninformed decisions about TOP. Their key concerns include decisions being made based on a NIPT result without diagnostic confirmation, with references to anecdotal accounts, and biased or uninformed perceptions of genetic conditions such as Down’s Syndrome (DS). Across the three countries, HCPs expressed concerns that we characterise here as NIPT leading women to make uninformed decisions around TOP, in the sense of not being fully informed whether about the test or the fetal condition. This is particularly relevant with regard to the possibility of earlier (and ‘easier’) access to TOP following NIPT.

## Comparison of the legal, regulatory and policy contexts

### Provision of NIPT and regulation of TOP in France, England and Germany

NIPT is available privately and offered as a publicly funded screening test in France (since 2019), England (since 2021) and on a case-by-case basis in Germany (since 2022). However, these countries define different thresholds and criteria for offering access to NIPT free of charge (see Table. 1). While France opted for a relatively low probability threshold (score ≥ 1/1000 in maternal serum screening (MSS)) making (expanded) NIPT available for a large group of women, England determined a higher threshold, limiting access to NIPT to a group of women with a higher probability (1:2 to 1:150) of having a child with T21, T18 or T13 following combined first-trimester screening (cFTS). Germany, on the contrary, decided to cover NIPT for T13, T18, T21, not based on a quantitative threshold, but on a case-by-case basis when the possibility of a trisomy strongly “burdens” the woman [19]. In the three countries, pregnant women can be prescribed NIPT by their gynaecologist-obstetrician, midwife or general practitioner. They may also be referred to a fetal medicine unit (FMU), a prenatal clinic or a medical genetics service offering NIPT.

**Table 1:** NIPT Policies in France, England and Germany

Year: NIPT publicly funded	France: since 2019 (expanded NIPT: since 2020)	England: since 2021	Germany: since 2022
Criteria for offering NIPT in each country	Second-tier screening test for increased probability of trisomy 21 (T21), trisomy 13 (T13) and trisomy 18 (T18) (score ≥1/1000 in maternal serum screening (MSS)) following combined first-trimester screening (cFTS) offered between 11 and 13 weeks of amenorrhoea OR fully reimbursed expanded NIPT for trisomies T2, T8, T13, T14, T15, T16, T18, T21 and T22, as well as large segmental aneuploidies when MSS test shows a score ≥1/1000.	Second-tier screening test for high probability (1:2 to 1:150) of having a child with Down's (T21), Edwards' (T18) or Patau's syndrome (T13) following combined first-trimester screening (cFTS) (11-14 weeks) or quadruple screening test (14-20 weeks).	Offered individually on a case-by-case basis, either when other tests (e.g. ultrasound, serum screening) have suggested a trisomy; or, when a woman together with her doctor come to the decision that the test is necessary in her personal situation. This situation can arise when the possibility of a trisomy so strongly burdens/distresses a woman that she would like to have it clarified.

Decision-making after a high probability (positive) NIPT result (and/or a confirmatory diagnostic test) is framed in the three countries by the governing bodies (HAS in France, NHS in England and G-BA in Germany) in terms of giving the pregnant woman the option of continuing or terminating her pregnancy. Although this paper focuses on termination of pregnancy for fetal anomaly, in this section we also describe the relevant legal frameworks, policy and regulation of TOP more broadly (see Table. 2), as TOPs after NIPT may also fall within gestational limits for access upon request.

**Table 2:** TOP Policies in France, England and Germany

Abortion law	France: Veil act (1975)	England: Abortion Act (1967)	Germany: Section 218a of the Criminal Code
Criterias for TOP access in each country	Time limit for TOP upon request(‘voluntary’ TOP (called 'IVG')): before 14 weeks (16 weeks of gestation). From 14 weeks and up to birth, a ‘medical’ TOP (called 'IMG') may be performed if two physicians who are members of a multidisciplinary team certify that: ‘the unborn child has a high-probability of suffering from a particularly serious condition recognised as incurable at the time of diagnosis’ (Article L2213-1 of the French Public Health Code).	*Ground A* : risk to the life of the pregnant woman (no time limit). *Ground B :* prevent grave permanent injury to the physical or mental health of the pregnant woman (no time limit). *Ground C :* risk of injury to the physical or mental health of the pregnant woman (before 24th week). *Ground D :* risk of injury to the physical or mental health of any existing child(ren) of the family of the pregnant woman (before 24th week). *Ground E : *risk that if the child were born it would suffer from such physical or mental abnormalities as to be seriously handicapped (no time limit). *Ground F : *to save the life of the pregnant woman (no time limit). *Ground G* : to prevent grave permanent injury to woman's physical or mental health	TOP permissible up until the 12th week after conception (14 weeks of gestation) with mandatory counselling and a 3-day waiting period. TOP permissible for ‘social-medical’ reasons at any gestation when deemed necessary ‘to avert a danger to the life or danger of a serious impairment of the physical or mental state of health of the pregnant women’.

### TOP in france: the veil act and constitutional change

In France, TOP is regulated under the so-called ‘Veil Act’ (named after its key proponent, Simone Veil) since 1975, and several legislative changes over the past decade have reduced restrictions on TOP access. TOP for ‘individual/social reasons’ (i.e. TOP upon request, or ‘voluntary’ TOP) until 14 weeks of pregnancy has been free of charge since 2013 to support the ‘fundamental right to abortion’. In 2014, the notion of a ‘distressing situation’ as a required justification for accessing ‘voluntary’ TOP was abolished to ensure ‘real equality between women and men’ [20]. The Bioethics Law of August 2021 removed the obligation to offer a woman a reflection period of at least one week before terminating her pregnancy if it is likely that the child will suffer from a particularly serious and incurable condition. In addition, in March 2024, France became the first country to enshrine the ‘guaranteed freedom’ to access ‘voluntary’ TOP in its constitution.

Further recent legal developments include the 2022 extension of the time limit for ‘voluntary’ TOP upon request by the woman and carried out by a physician or specialised midwife from 12 to 14 weeks of pregnancy [21]. This coincides with the period during which NIPT is performed. The 2022 law states that, because access to TOP is a ‘right’, women do not have to provide any justification for their decision to HCPs. Beyond 14 weeks of pregnancy (i.e. 16 weeks since the last period), a ‘medical’ TOP may be performed at any point if two physicians who are members of a multidisciplinary centre for prenatal diagnosis certify that ‘*the unborn child has a high probability of suffering from a particularly serious condition recognised as incurable at the time of diagnosis*’ (Article L2213-1 of the French Public Health Code).

### TOP in england: legislation that is interpreted broadly in practice

Unlike in France, in England, women are not eligible for TOP without approval by two independent physicians, and there is no distinction between unconditional (‘voluntary’) and conditional (‘medical’) TOP. In England, TOPs are lawful, i.e. exempt from punishment, under specific conditions regulated by the UK Abortion Act 1967 (as amended by the Human Fertilisation and Embryology Act 1990). If these conditions are not complied with, women and/or physicians might risk being prosecuted for criminal offence. The law distinguishes between TOPs up to 24 weeks and TOPs from 24 weeks up to birth. Up to 24 weeks, physicians have the option of recording TOP for fetal anomaly (TOPFA) on the ground of the pregnant woman’s mental or physical health or on the ground of severe fetal anomaly. However, the ‘mental/physical health’ ground may be interpreted very broadly in practice, resulting in *de facto* access to abortion upon request in many areas [22]. In England and Wales, 98%, of TOPs (247,440) were performed on this ground in 2022 [23]. Where a pregnant woman may want to terminate her pregnancy following a positive NIPT result before 24 weeks, this request can be granted with reference to the woman’s wellbeing, rather than with a diagnosis of fetal anomaly. There was a call in 2023 by the Royal College of Obstetricians and Gynaecologists and 32 organisations to reform the Abortion Law and, in particular, to remove the requirement for two doctors’ signatures to authorise an abortion [24]. The call has not resulted in a reform thus far but UK MPs are set to vote on whether to decriminalise abortion in England and Wales this summer (2025) [25].

### TOP in germany: compulsory reflection and mandatory counselling before TOP

The German case [26] offers an interesting point of comparison with the English and French legal and regulatory frameworks. TOP is regulated under the German Criminal Code (Sect. 218, 218a-c, 219, & 219b StGB). It is permitted upon request until 12 weeks after conception (14 weeks since the last period) following mandatory ‘pregnancy conflict counselling’ and within a three-day waiting period between the counselling and the procedure. The counselling is intended to ‘help [the pregnant woman] to make a responsible and conscientious decision’ about her pregnancy, while also aiming ‘to protect the unborn life’ and ‘to encourage the woman to continue the pregnancy’ (Article 219 StGB). While in France and the UK, TOP can be accessed through the public healthcare system, in Germany statutory health insurances may only cover some associated costs (e.g. pre-procedure consultations). For non-medical or criminological indications, women have to pay for the TOP procedure itself (up to 12 weeks of conception between 300 and 600 EUR), unless they are eligible for social aid when it is covered by the state [26].

In addition to the ‘individual or social’ exception to perform TOP up to 12 weeks (14 weeks of gestation), TOP may be exempt from punishment at any gestation ‘to avert a danger to the life of or the danger of grave impairment to the pregnant woman’s physical or mental health’ (Section 218a StGB). In Germany, diagnosis of a fetal anomaly does not constitute a legal ground for TOP to avoid value judgement about ‘disabled life’, yet in practice, the ‘medical’ indication covers the potentially serious effects of a diagnosis of health condition in the ‘unborn child’ on the woman’s wellbeing. If there is an ‘impairment to the child’s physical or mental health’ [27] detected during prenatal testing, the medical indication requires comprehensive and non-directive counselling (‘ergebnisoffen’) [28] about the condition which ‘leaves the decision open’. Like in TOPs up to 12 weeks, there is a three-day compulsory reflection period between the communication of the diagnosis and the written determination of medical grounds for terminating the pregnancy [27]. Although the German Medical Association provides a list of doctors, hospitals and establishments providing TOP, in practice, reproductive health organisations (e.g. profamilia) in Germany have highlighted the difficulty for women to find physicians who will issue an indication, and to find a physician or a clinic that performs an abortion in the second or third trimester (knowing that the physician issuing the indication and the one performing TOP cannot be the same). The number of clinics reporting abortions decreased 46.7% between 2003 (2,050 clinics) to 2021 (1,092 clinics), with some regions experiencing a disproportionate shortage [29]. The costs of TOP performed on medical ground are covered by the statutory health insurance funds.

### Reading TOP statistics from a NIPT perspective

There is no established evidence of a clear relationship between the introduction of NIPT and any changes in the rate or indications of TOPs in the three countries. Previous research has found minimal evidence for an association between NIPT and TOP or birth outcomes [30]. Furthermore, complicating factors for the period that covers the introduction of NIPT into the English, French and German public healthcare systems include the COVID-19 pandemic and associated events; total birth rates; changes in TOP legislation; and changes in population size or demographic factors such as maternal age.

In France, there was an increase in the number of ‘voluntary’ terminations in 2022 (232,000); however, this followed a decrease in 2020 and 2021 compared to 2019 (224,000), which has been attributed to the COVID-19 pandemic [31]. Figures do not indicate how many ‘medical’ TOPs are carried out by French women after receiving authorisation to have a TOP. However, there was a slight increase in the number of attestations of ‘particular gravity’ (fetal indication) delivered by multidisciplinary centres for prenatal diagnosis (CPDPN) between 2018 (6754) and 2019 (7067) around the time that NIPT was introduced in France in January 2019 [32]. The number of chromosomal indications identified and reported by CPDPN also increased during this period, rising from 2767 (2018) to 3146 (2019) [32].

In England (and Wales), where TOPFAs tend to be recorded under the ground of the pregnant woman’s mental or physical health rather than on the ground of severe fetal anomaly before 24 weeks, the use of the woman’s wellbeing indication has increased since the introduction of NIPT in 2021 (from 209,939 to 247,440 in 2022) [23, 33]. However, there is no evidence to suggest that this is because of increased detection of fetal abnormalities. Financial factors and difficulties in accessing contraception are among the main reasons given by some commentators [34].

Finally, in Germany, although data on abortion is less comprehensive than in England or France, since NIPT is covered by the health insurances (2022), TOP rates altogether appear to have risen: an increase of 9.9% in 2022 to around 104,000 cases from the level of 2021 (around 95,000 cases) and an increase again in 2023 compared with 2022 by 2.2% (around 106,000 cases) [35]. However, TOP rates remain low compared to France, England and Wales. The increase in Germany has led some commentators to make the link with the availability of NIPT through the public health system [35], but there is a lack of comprehensive data from Germany.

Given the lack of evidence, it is currently impossible to make any claims about proposed causal effects of NIPT on pregnancy termination rates and/or indications in France, England and Germany, despite the significant uptake of NIPT in these three countries.

## Methods

This present paper reports on findings from a cross-cultural comparative qualitative interview study with French, English and German HCPs. Our empirical approach also builds on a comprehensive literature review [36] in French, English and German focusing on the regulations and arguments concerning NIPT and abortion (legal and regulatory texts; public reports of national ethics committees and professional bodies; parliamentary debates; medical press; academic literature in prenatal genetics, bioethics, social sciences; and daily press). In this paper, we focus on HCPs perspectives and concerns on TOP following a positive NIPT result, i.e. discussions about TOP with women/prospective parents and their approach, understanding, perceptions, fears and concerns about TOP after the use of NIPT (and when relevant, a diagnostic test).

## Data collection: semi-structured interviews

The inclusion criteria were HCPs who are involved with, have experience with, or have other relevant knowledge regarding the provision of NIPT at various stages of testing, including post-test counselling and return of results, in France, England or Germany.

We recruited 58 HCPs for semi-structured interviews: 23 in England, 17 in France and 18 in Germany (see Table. 3). At the time of data collection, some of the interviewees were not themselves practicing in England or France (1 from Wales, 1 from Scotland, 1 from Belgium), but had previous experience and/or expertise relevant to these contexts.

**Table 3:** Participant characteristics (HCPs)

	France = 17	England = 23	Germany = 18
Obstetrician-Gynaecologists	4	1	6
Clinical Geneticists	5	4	
Genetic Counsellors		5	
Prenatal Medicine Specialists		2	6
Midwives	5	6	
Nurse		2	
GP	1		
Laboratory/Medical Biologists	2	2	
Pregnancy/Prenatal Counsellors			6
Doctor & Medical Ethicist		1	

The recruitment was undertaken by AP, HBS and RH. HCPs were recruited through pre-existing professional networks within prenatal genetics and policy, followed by subsequent snowball sampling. The 58 semi-structured interviews were conducted in French, English and German online via Microsoft Teams by three qualitative researchers, AP, RH and HBS, between July 2021 and February 2023.

The interviews were of approximately 45 min duration each. The interviews were digitally recorded and transcribed verbatim.

The interview guide was not specifically thematised to focus on the issue of TOP, but this salient theme did emerge during the interviews. Through their responses to more general questions around NIPT (such as experiences with the implementation and/or provision of NIPT; attitudes and opinions towards NIPT; views about decision-making and the counselling process), HCPs addressed topics such as perceptions related to women’s attitudes, knowledge and decisions regarding TOP; perceptions about legitimate reasons for having or not having a TOP; perceptions related to the psychological and relational consequences of TOP.

### Interview analysis

Following a thematic analysis approach [37], the interviews were coded first separately and then cross-coded by AP, HBS, TNK and RH. The French, English and German interviews were initially analysed as three discrete datasets and then brought together for comparative analysis. The collaborative coding involved regular meetings between the researchers to discuss and review the construction of the meaning of the codes, in general, and with regard to their cultural and linguistic translatability, and their applicability to each group of participants. By doing so, a master codebook was developed and then in turn applied to the interview data using NVivo software. Data were de-identified to protect the privacy of interviewees while retaining context and content as much as possible.

## Results

In this section, we present two concerns highlighted by HCPs in France, England and Germany regarding women or prospective parents’ decision-making about TOP following a positive NIPT result. Through our interviews, HCPs explored key fears and concerns arising from their own experience in clinical practice, the reported experiences of colleagues, and broader social and political discourse. HCPs raised two key concerns: that women may make decisions without a confirmed diagnosis and that they may have a biased perception of the genetic condition screened for, such as DS.

Critically, the data we present here should not necessarily be interpreted as evidence *supporting* their fears and concerns. Rather, our examination is of the nature and focus of their perceptions.

### Fears of hasty decisions around TOP

In each of the three countries, HCPs expressed fears that women and prospective parents may make hasty or “panic”-driven decisions about TOP based solely on NIPT results, without confirming any findings through diagnostic testing (CVS and amniocentesis are carried out from 11 to 15 weeks respectively). This could lead to women making an insufficiently informed or uninformed decision about the course of their pregnancy. However, no HCPs detailed specific instances where this had occurred in their own clinical practice. Some made general references or focused on it as a possibility, or how this fear influenced their counselling of patients; many also described how they had heard anecdotal or second-hand reports of this phenomenon occurring (e.g. from colleagues).

During the interviews, HCPs frequently referenced the gestational timing of NIPT, and how this could allow women or prospective parents to opt straight for a TOP upon request in many jurisdictions. This was overwhelmingly framed as a negative potential choice and something that must be avoided. Many indicated that one aspect of their role as clinicians was to prevent this occurring, by effective counselling of patients.

#### Concerns about unconfirmed diagnoses and the extension of the legal limit for ‘voluntary’ TOP in France

Some French HCPs [4] described difficulties in convincing women of the importance of confirming a NIPT result through an invasive diagnostic procedure (CVS or amniocentesis). They expressed concerns that some women would like to have a TOP based on an initial NIPT result rather than having to wait for the diagnostic test.

‘(…) what is sometimes complicated to pass on to them is that this additional diagnostic stage is necessary (…) i.e. there are some who would like to terminate their pregnancy straight away on the basis of the first result and this is sometimes complicated to manage’ *[French HCP 17_ Professor in Genetics]*.

A number of French participants referred to the extension of the legal limit for TOP upon request (‘voluntary’ TOP) - from 12 to 14 weeks of pregnancy (16 weeks after the last menstrual period). They raised the possibility that this could broaden the period for women to seek TOP immediately after NIPT. At the time of the interviews, HCPs were aware of the plan to extend the legal limit on TOP upon request.

‘(…) on serum markers or NIPT, if patients manage to have them before 14 weeks, no one can prevent them from going for ‘voluntary’ TOP. Nobody can stop them.’ *[French HCP 12_ Midwife]*.

Another participant, a French gynaecologist, described fears that some women (and couples) may impulsively decide to undergo a TOP before 16 weeks of amenorrhoea, while there is still uncertainty (no diagnostic confirmation) about the significance of the positive result, which is exacerbated in the case of a rarer condition (e.g. rare trisomy, large deletion or duplication) detected by expanded NIPT:

‘Personally, I’m having great difficulty with this extension of the time limit, because a lot of tests [expanded NIPT] are carried out during this period, (…). And where women and couples panic and can then choose to erase everything and start again.’ *[French HCP 11_Obstetrician-gynaecologist]*.

Respondents described the challenges involved in examining requests for TOP on ‘medical’ grounds for rarer chromosomal abnormalities (rare autosomal trisomies, large deletions and duplications) in terms of the complexity of their interpretation (e.g. varying degrees of phenotypic expression, lower accuracy).

‘After that, there is also the whole question of, (…), there are chromosomal abnormalities [beyond the three common trisomies] that are not always going to be serious, not always admissible for a TOP, (…) and so, we expect to be faced with ethical difficulties’. *[French HCP 8_Geneticist]*

This interviewee underlines the dilemmas faced by HCPs when judging the eligibility of TOPs for rarer (and potentially less serious) chromosomal abnormalities.

#### Concerns about unconfirmed diagnoses and lack of follow up in the private sector in England

The English interviews focused on concerns about provision of NIPT in the private sector, and the quality of test provision and clinical counselling. Some interviewees [8] described cases (whether of their own or colleagues) of women who had carried out NIPT in the private sector and then turned to the public sector (NHS) for more genetic advice on the chromosomal abnormalities screened beyond the three trisomies (T21, T13 and T18).

‘Colleagues of mine (…) have had similar issues where actually NIPT result [in the private sector] has been wrong and the couple have, through the NHS, after they’ve been given a result, then had an invasive test. (…), and in most situations where they might be having a termination based on that result. That’s obviously going to be vital that that result has been confirmed. Because they could be making a decision based on an incorrect result.’ *[Eng HCP 19_Genetic Counsellor]*.

In addition, this English nurse describes situations where she believes that women would terminate their pregnancy without diagnosis:

‘Sadly, I’ve heard of cases of terminations just on NIPT without any confirmation. I don’t think I’ll be alone there and that seems wrong.’ *[Eng HCP 3_Nurse]*.

The following interview highlights the perception of a lack of follow-up in the private sector after the return of the screening results, and the risk of making a decision without receiving proper counselling and nuanced information about the detected condition. Some English HCPs [9] also described concerns about the uncertainty of the variability (either as penetrance or expressivity) of other conditions that may be offered by the private sector.

(…) there’s this bigger push… some of the private NIPT companies are now looking at things like 22q deletion and other conditions. I think we do need to be careful as to how far we go with offering tests, particularly for very variable conditions that lots of people with them have a very good quality of life, (…) And there are still people who say: ‘Well, if my baby’s got a genetic condition, I want a termination,’ but of course there are thousands of genetic conditions and some are truly awful and some are much milder, and it’s very difficult. *[Eng HCP 010_Geneticist]*

English HCPs in our study were concerned that the provision of NIPT in the private sector may lead women to consider TOP based on insufficient clinical knowledge of the conditions being screened for (e.g. phenotype at birth), which could potentially result in TOP for less serious or non-serious conditions.

#### Concerns about early TOP without diagnosis due to gestational restrictions in Germany

The German interviewees emphasised the concern that women might decide to terminate their pregnancy early – before 12 weeks – having received neither genetic counselling nor a diagnostic confirmation. This reflects in particular the time pressure faced by women in Germany and the concern that women will make early TOP decisions due to the difficulty of accessing TOP in Germany after 12 weeks.

‘(…) I’ve also heard this from colleagues – that women use the test (…) in the very early weeks of pregnancy without having much knowledge about it [NIPT]. And that possibly before the 12th week of pregnancy in Germany, an abortion is made (…).’ *[Ger HCP 3_ Pregnancy/prenatal counsellor]*.

(…) it is actually the case that if the women really want to have an abortion after this [NIPT] finding and then without medical indication, just get [the abortion] based on NIPT without further [diagnostic tests] (…). [*Ger HCP 11*_ *Gynaecologist*]

In the following two interviews, German HCPs place particular emphasis on the risk of making an uninformed decision to terminate the pregnancy of a ‘healthy child’ without confirmatory diagnosis:

‘But again, if a woman decides to have an abortion based on a positive NIPT, then there’s a 50% chance that she’ll abort a healthy child if she’s under 30, unless the child has something what is seen on ultrasound. But if I don’t do an ultrasound, I can’t tell.’ [*Ger HCP 21_ Doctor-prenatal medicine specialist*].

‘The job [of counselling] is for them to make it clear that such a result is not 100%, that it is not a diagnosis in that sense and that you are taking the risk of possibly terminating the pregnancy of a healthy child, you see?’ *[Ger HCP 16_ Obstetrician-gynaecologist]*.

German HCPs stressed the importance of counselling to avoid terminating the pregnancy of a ‘healthy child’.

Across the three countries, interviews with HCPs highlighted a range of concerns about women taking insufficient time for deliberation or making decisions about TOP. They focused on the importance of discussing with women the implications of the condition detected and of confirming a positive NIPT result with further diagnostic testing before considering TOP.

### Concerns about media representations and social beliefs relating to DS

Across the three countries, HCPs raised concerns that women or prospective parents may not have an accurate, balanced or realistic picture of the impact of DS on life – i.e. how people and their families can actually live with disability – and that this could have an impact on informed decision-making. In France and England, HCPs expressed reservations about media representations of disabilities such as DS, which focused only on those who were independent, and doing well. They felt these representations did not sufficiently reflect the full range of scenarios, such as the medical complexity of the condition or potential challenges that families might face. In contrast, in Germany, when discussing the need for a balanced and realistic representation of DS in counselling, HCPs focused much more on avoiding overly *negative* portrayals of quality of life. They emphasised the importance of communicating to patients the rich and full life that children with disabilities such as DS can lead.

#### Concerns about media representations of disability in France and England

HCPs in France and England made references to representations of DS in the media. They felt that these did not sufficiently reflect medical aspects of a condition like DS (e.g. cardiac problems, likelihood of developing certain health conditions), which was important for prospective parents to know in order to make informed decisions. They described how these media representations might lead to ‘false perceptions’, and/or how they might not validate the realities of parents with children who were not independent or ‘doing well’.

A few French HCPs referred to the television campaign, ‘Dear Future Mom’, which was broadcast across Europe on World Down Syndrome Day (YouTube, 21 March 2014). The clip was produced by 15 European Down’s syndrome associations. It features 15 children of different nationalities describing their lives with DS, and in particular their abilities, which are portrayed as similar to those of other children.

‘There’s been a kind of [television] campaign where you see more children with DS on television, with children who are relatively independent, who seem to be doing well in the end in well-off families. (…) Afterwards, we talked mainly about children with DS who were doing well, in other words who were independent. We didn’t talk about those with heart defects. We didn’t talk about those who never spoke and who had severe autistic syndromes.’ *[French HCP 12_ Midwife]*.

In England, the BBC documentary, ‘A World Without Down’s Syndrome?’ [38] presented by Sally Phillips, in October 2016, was mentioned multiple times by English HCPs. Sally Phillips is an English actress who has a son with DS. She made the documentary to portray the positive aspects of having a child with DS, and critique the possible implementation of NIPT in the NHS and the risk of it ‘eradicating’ the DS population.

‘There’s sometimes quite a false perception in that you’ve got somebody who’s an actress [Sally Phillips] who can really shout loudly: ‘look at me and look at what my child’s achieved!’, but there was nobody there [in the meeting] that had a child with DS that had really struggled with lots of health problems.’ *[Eng HCP 6_Genetic Counsellor]*.

HCPs in France and England focused on concerns that women may base their decisions on positive media or popular views of DS that may be a ‘false perception’ of what it is like to live with the condition. They framed this primarily in terms of lack of emphasis on medical aspects of the condition (e.g. some level of learning disability, a heart condition in about half cases, and other health conditions that are more common in people with DS), and that these portrayals of DS risk creating a sense of guilt among women considering having a TOP. A focus on the medical aspects of disability in prenatal counselling has been subject to significant critique in the bioethics and disability literature [39, 40]. This includes descriptions of conditions such as DS primarily in terms of medical symptoms and framing it in terms of deficit, and how this can impact the choices made [40]. However, media representations of disability that aim to be positive and counter medicalised and hurtful narratives also risk obscuring lived realities and stereotyping people with DS, which includes difficulties in access to services and resources [41].

#### Countering negative beliefs or perceptions of the ‘worst-case scenario’ in Germany

In comparison to the French or English interviewees, German HCPs stressed fears that women decide to terminate their pregnancy based on overly negative perception of disability, and a lack of understanding of the quality of life that a child with DS might have. German HCPs described how some women imagine the ‘worst-case disability’ *[Ger HCP 3_Pregnancy/prenatal counsellor]* when they receive the result indicating a fetal condition, which may be because they do not know anyone living with this condition in their social circle or environment. While French and English HCPs made reference to specific media representations, German HCPs focused more on general social perceptions of disability, lack of understanding of genetic conditions, and the unnecessary equation of disability with ‘suffering’.

‘Then there are also a lot of ideas that disability means suffering. (…) And I don’t think they have any idea how satisfied people with DS can be with life’. *[Ger HCP 7_ Pregnancy/prenatal counsellor*,* systemic therapist]*

‘(…) DS in its psychological and physical developmental variants does not necessarily have to be equated with a serious illness. The prenatal diagnosis results in a possibly insufficient knowledge about the condition or the clinical picture and the consequences in a rapid TOP.’ [*Ger HCP 18_Doctor-prenatal medicine specialist]*.

German HCPs expressed concern that women may decide to terminate a pregnancy although it is difficult to determine how severely a child might be affected by DS.

‘DS, the spectrum is extremely large, without us being able to say in advance. That only becomes apparent after the birth and there are certainly a number of people with DS working here at the clinic, in the emergency room as nurses, in the kitchen. They also live independently. They come here to the clinic on their own by tram and can lead a very contented, self-determined life.’ *[Ger HCP 27_ Doctor-prenatal medicine specialist]*.

Although they acknowledge the variable expressivity of the condition, several HCPs interviewed particularly emphasised the fact that some people with DS can live independent lives.

‘So, if you knew now that all people with DS have a slight mental impairment, but are otherwise happy people and can eventually live relatively independently and do everything (…)’ [*Ger HCP 10_Doctor-prenatal medicine specialist*].

The German interviewees mentioned the importance of offering genetic counselling sessions so that women have a more ‘realistic’ view of a disability such as DS and the possible resources and support for raising a child with a disability.

To summarise, French and English HCPs in this study were concerned about women being influenced by overly positive media representations in their decisions about the course of their pregnancy. German HCPs, on the other hand, feared that women have an overly negative and biased view of life with a condition such as DS. They tended to emphasise the unknown severity or variable expressivity of the disease, as well as the potential independence and happiness of people living with it.

## Discussion

In this section, we critically analyse and compare HCPs’ concerns about what they perceive as the risk for women or prospective parents to make uninformed decisions about TOP following a high probability (positive) NIPT result in France, England and Germany. The specific nature of these concerns was framed differently in the three countries reflecting the social, policy and legal context, but can be drawn together by two underlying currents or interpretations. The first is about the test - that women may make an uninformed decision about the significance of NIPT results due to the lack of understanding of the test’s technical shortcomings and importance of diagnostic confirmation, and that the timeframe of TOP upon request might lead to ‘panic’ and hasty decision-making around TOP. The second relates to the fetal diagnosis – the concern that women may make an uninformed decision about the condition identified in the fetus due to an unrealistic or false perception of the disability such as DS, in relation to what is perceived as media and popular representations in France and England or negative societal beliefs around disability in Germany. DS was likely a focus in the interviews because it is one of the most common autosomal aneuploidies, and characterised by higher live birth rates compared with other conditions screened for such as T18 and T13 [42].

While ‘informed choice’ is a central concept in prenatal screening, it can be understood in a range of ways, and there is a lack of consistency in how this construct can be measured in clinical practice and utilised in policy [43]. In the prenatal setting, there can be multiple factors involved; a measure by Lewis et al. [44] focuses on the domains of knowledge, attitude and deliberation. The knowledge required for decision-making around TOP may be understood in two broad senses: a full and correct understanding of the nature of the test and test results; or, having access to the most accurate and useful information about their particular case (i.e. a diagnostic confirmation). Depending on how we understand informed choice, where a woman makes a decision to undergo TOP based on a high probability NIPT result, fully aware that it is *not* a diagnostic confirmation but only a probability, she may still well have made an ‘informed choice’. Some women may accept this uncertainty and still be willing to have a TOP without a diagnostic test, given their personal and family circumstances and/or their preference for an early TOP [45]. This is particularly the case where gestational limits on access to TOP may, even if only in theory, affect their ability to access TOP services after confirmation of results through prenatal diagnosis. Elsewhere, we have explored how the 12-week limit in Germany may potentially place pressure on women to access TOP early, before access to TOP on medical grounds becomes more complicated [46].

Even in circumstances where women and prospective parents may lack information or understanding about critical aspects of the test or fetal diagnosis, we would like to emphasise that they may still feel sufficiently informed to make a decision and an autonomous choice in the light of their preferences, values and beliefs. This may lead them to make a pragmatic choice and suboptimal decision, i.e. the least bad decision possible given their individual situation. In these cases, women would be making a decision based on the balance of probabilities of two undesirable scenarios: the probability that they will terminate a pregnancy *without* a fetal anomaly, versus the probability that the fetal anomaly will be confirmed but they will be unable to access an (earlier, and possibly ‘easier’) TOP, and maybe struggle to be eligible for a later TOP. TOP before 12/14 weeks involves a less invasive procedure for the woman against later TOP, which involves the use of general anaesthetic or sedation, and of vacuum aspiration or forceps. Several authors have stressed the importance of early TOP in limiting the psychological impact, risk of depression and harm to women, as well as for health safety and costs [47]. At this point, we would further like to stress that we have little to no data or evidence on the actual rates or incidence of women making decisions about TOP based solely on a NIPT result. Our data here report on HCPs’ concerns based on what appear to be second-hand reports or their own interpretations of women’s attitudes and potential choices.

As shown in the results section, HCPs raised concerns – which have also been raised in the clinical literature [48] – that women are considering or deciding to have a TOP without diagnostic confirmation of the NIPT result and the fetal condition. HCPs framed this as a potential negative phenomenon, and to counteract it they described how they stressed the importance of diagnostic confirmation in their counselling processes. While our previous research explores how HCPs place values such as choice, autonomy and freedom as central in their counselling more broadly [3], the particular question of decision-making around TOP draws out more paternalistic or interventionist perspectives about a duty to prevent uninformed or inappropriate decision-making.

Because of the non-diagnostic nature of a screening test diagnostic testing (amniocentesis or CVS) is recommended as an important step before TOP following NIPT. NIPT requires follow up of positive cases to determine if they are false or true positives.

A 2022 meta-analysis estimates that in a general population, the positive predictive value (proportion of true positives from all positives) was 91.78% for trisomy 21 (DS), 65.77% for trisomy 18 (Edwards syndrome), and 37.23% for trisomy 13 (Patau syndrome) [49]. It is important to note that the positive predictive value of a screening test varies based on its prevalence in the population, and thus increasing the scope of an NIPT offer to all pregnancies can impact this value, which is related to the individual’s a priori risk based on factors such as maternal age. However, false positives (FPs) can be assessed by other measures, which illustrates the complexity of communicating this information. For example, while the majority of positive results for trisomy 13 are likely to be FPs (as seen in the lower positive predictive value), the false positive rate in unaffected pregnancies for trisomy 13 is 0.04%. There is a risk that the potential of NIPT will be misrepresented or misunderstood depending on how information about FPs is communicated. HCPs have the responsibility to provide women and prospective parents with comprehensive data on FPs and to explain accurately the limitations of NIPT [50]. This responsibility may underly the feelings of concern that HCPs in our study described about the risk of proceeding to TOP without diagnostic confirmation.

There is a strong emphasis in clinical practice in all three countries on the importance of diagnostic testing following a positive NIPT result. The insistence on the diagnosis can be seen as a means of avoiding the risk of harm to women due to the uncertainties and complexities of interpretation generated by prenatal screening [51], particularly with the development of whole genome sequencing [52, 53]. While this approach to the concept of informed decision from a medical perspective is important, it is also likely that other factors (psychosocial, medical, and economic) related to the circumstances of women and prospective parents come into play when deciding on the course of a pregnancy. These factors can broaden the concept of informed decision in the medical field by taking into account the realities experienced by women and prospective parents. This means that, in addition to the medical information that patients receive during counselling, the decision-making process should also incorporate the social and personal aspects of choices [54], i.e. the balance of arguments according to which patients feel they have made well informed decisions regarding their circumstances (e.g. gestational age, mental health issues, worries, relational and work problems, etc.).

Many clinical guidelines or policies also emphasise the importance of non-directiveness, providing balanced information and supporting women to make the decision that is right for them [55, 56]. However, our research illustrates some of the difficulties in meaningfully applying this concept in day-to-day clinical practice. The concept of non-directivity is often understood at a semantic or explicit level. Our study shows that it is difficult to apply non-directivity in socio-cultural and legal contexts where certain orientations have already been taken and norms are already in place. For example, there are significant contrasts between cultural models such as the overload of medical information surrounding DS in France [3] and the focus on information about quality of life with DS in Germany. Different values come into play in the attempt to convey balanced information during clinical consultations. In the three countries, non-directiveness seems to be shaped by cautious approaches to risk: this may be fear of legal action in France (e.g., fear of litigation if a genetic condition in the fetus is not detected); respect for the rights of people living with disabilities in England; and concerns about eugenics in Germany. These values and concerns are present in all three settings, but the emphasis and interpretation differ. The values implicitly conveyed by HCPs reflect the cultural settings in which they are practising. It would be important for HCPs to consider how to take a step back from potential cultural biases and to recognise how socio-cultural and legal contexts can influence counselling styles.

For example, in Germany, genetic counselling following the detection of a genetic condition is seen as a necessity for women to be well informed when considering TOP for ‘medical’ reasons [27]. Professionals are required to provide detailed information about the medical, social and psychological aspects of the diagnosed condition [27], and are expected to involve those who provide follow-up care for children born with the condition. In addition, they are required to provide pregnant women with information about support available for caring for a child with special needs, and about self-help groups and disability associations [27]. Pregnant women also receive this information in France and England (information on conditions, list of disability associations), but perhaps with less details and less authority on the obligation to receive this type of information if the woman requests a TOP, particularly in England where counselling is conceived as being at the request of the woman.

Due to its Nazi past, there is a prominent fear in Germany of making value judgements about life with disability and discriminating against it [57, 58]. This reflects the tension in Germany between the principle of reproductive self-determination and the principle of inclusion of disability in society [58, 59]. As explored in this study and elsewhere in the literature, this tension is less prominent in England and France.

HCPs in France and England focused more on concerns that women may base their decisions on positive media or popular views of DS that may be a ‘false perception’ of what it is like to live with the condition. They framed this primarily in terms of lack of emphasis on medical aspects of the condition (e.g. some level of learning disability, a heart condition in about half cases, and other health conditions that are more common in people with DS), and that these portrayals of DS risk creating a sense of guilt among women considering having a TOP. However, a focus on the medical aspects of disability in prenatal counselling has been subject to significant critique in the bioethics and disability literature [39, 40]. This includes descriptions of conditions such as DS primarily in terms of medical symptoms and framing it in terms of deficit, and how this can impact the choices made [40]. However, media representations of disability that aim to be positive and counter medicalised and hurtful narratives also risk obscuring lived realities and stereotyping people with DS, which includes difficulties in access to services and resources [41].

## Conclusion

In France, England and Germany, the provision of NIPT can give women or prospective parents access to more information about their pregnancy but also raises concerns among HCPs about the quality of this information, and the fear that this could lead to women deciding to have a (early) TOP without confirmatory diagnosis or adequate understanding of the associated condition. In this paper, we have highlighted similarities between the concerns of French, English and German HCPs that women would make an uninformed decision following a high probability NIPT result. Women might undergo unwarranted TOPs (TOPs without diagnosis) and/or make a decision based on a biased perception of DS. Our results also show differences, mainly in the concerns expressed by German HCPs about the negative perceptions women or prospective parents may have of DS and how it can influence their decision to have a TOP. Although aware of the wide spectrum of DS, German HCPs place more emphasis on the quality of life and ‘happiness’ with the condition than French and English HCPs.

Through their concerns, French, English and German HCPs tend to project what should be the ‘right approach’ for pregnant women or prospective parents: how to make an appropriate decision about TOP following a high probability (positive) NIPT. Informed choice may be best facilitated by access to the most certain or comprehensive information – i.e. results from diagnostic testing. We want to clearly emphasise the duty professionals have to duly inform women about the technical shortcomings of the test. However, we should also acknowledge the view that women are capable of making informed decisions themselves if they have received sufficient information about the limitations of NIPT and the condition being screened. Empirical studies support that they are capable to determine the level of uncertainty they are willing to tolerate [60] when considering access to TOP in (early) pregnancy, and determine their own view of the disability [61] and the potential severity associated. Policies and medical practices aimed at protecting pregnant women from the risks of harm related to prenatal testing could inadvertently lead to paternalistic approaches or attitudes if they do not take into account women’s experiences and their individual situations [2].

## Data Availability

Data are available from the UK Data Archive for researchers who meet the criteria for access to confidential data. Horn, Ruth (2023). Non-invasive Prenatal Testing Study: Comparison England, France, Germany, 2021-2022. [Data Collection]. Colchester, Essex: UK Data Service. 10.5255/UKDA-SN-856508 https://reshare.ukdataservice.ac.uk/856508/.
